# Potential inhibitory effects of compounds ZK-PI-5 and ZK-PI-9 on trehalose and chitin metabolism in *Spodoptera frugiperda* (J. E. Smith)

**DOI:** 10.3389/fphys.2023.1178996

**Published:** 2023-03-29

**Authors:** Fan Zhong, Liuhe Yu, Xinyi Jiang, Yan Chen, Sitong Wang, Lei Chao, Zhiyang Jiang, Biner He, Caidi Xu, Shigui Wang, Bin Tang, Hongxia Duan, Yan Wu

**Affiliations:** ^1^ College of Life and Environmental Sciences, Hangzhou Normal University, Hangzhou, China; ^2^ Innovation Center of Pesticide Research, Department of Applied Chemistry, College of Science, China Agricultural University, Beijing, China; ^3^ Guizhou Provincial Key Laboratory for Rare Animal and Economic Insect of the Mountainous Region, Department of Biology and Engineering of Environment, Guiyang University, Guiyang, China

**Keywords:** Spodoptera frugiperda, trehalose metabolism, chitin metabolism, trehalase inhibitor, pest control

## Abstract

**Introduction:** *Spodoptera frugiperda* is an omnivorous agricultural pest which is great dangerous for grain output.

**Methods:** In order to investigate the effects of potential trehalase inhibitors, ZK-PI-5 and ZK-PI-9, on the growth and development of *S. frugiperda*, and to identify new avenues for *S. frugiperda* control, we measured the content of the trehalose, glucose, glycogen and chitin, enzyme activity, and gene expression levels in trehalose and chitin metabolism of *S. frugiperda*. Besides, their growth and development were also observed.

**Results:** The results showed that ZK-PI-9 significantly reduced trehalase activity and ZK-PI-5 significantly reduced membraned-bound trehalase activity. Moreover, ZK-PI-5 inhibited the expression of *SfTRE2, SfCHS2,* and *SfCHT*, thus affecting the chitin metabolism. In addition, the mortality of *S. frugiperda* in pupal stage and eclosion stage increased significantly after treatment with ZK-PI-5 and ZK-PI-9, which affected their development stage and caused death phenotype (abnormal pupation and difficulty in breaking pupa).

**Discussion:** These results have provided a theoretical basis for the application of trehalase inhibitors in the control of agricultural pests to promote future global grain yield.

## 1 Introduction

Trehalose, as a reducing sugar, is widely present in bacteria, fungi, plants, and invertebrates. It was first extracted by Wiggers from corn with *Claviceps purpurea* (Wiggers, 1832). Trehalose mainly exists in all stages and almost all tissues of insects (Wyatt, 1967; Elbein et al., 2003). More studies have shown that trehalose is of great significance to insect growth, development, molting, and metamorphosis (Elbein et al., 2003; Tang et al., 2014; Tang et al., 2017). Trehalase is divided into soluble (TRE1) and membrane-bound trehalase (TRE2), and is a glycosidase that is responsible for hydrolyzing trehalose into two glucose molecules (Tang et al., 2017; Tang et al., 2018). Trehalose is called insect blood sugar, which is regarded as an alternative and standby energy source of glucose and faced with stress condition (Thorat et al., 2012). As an immediate energy source, it plays a vital role, such as providing energy during flight (Elbein et al., 2003; Shukla et al., 2015). Trehalose is hydrolyzed glucose to provide energy for the biological organism (Wang et al., 2020). In this process, due to high specificity for trehalase, it is competitively inhibited by most glycosidase inhibitors (Shukla et al., 2015; Tang et al., 2018). Trehalase inhibitors have been shown to improve the mortality of insects during their growth and development (Wegener et al., 2003; Forcella et al., 2010; Tang et al., 2018). Trehalase inhibitors directly reduce trehalase activity, thus affecting trehalose hydrolysis and chitin synthesis during insect molting (Chen et al., 2010). Owing to the dependence of insects on trehalose and the key role of trehalose enzymes in insect carbon metabolism, trehalase inhibitors may have broad applications in pest control (Shukla et al., 2015).

Chitin is an important component of the epidermis, trachea, and midgut peritrophic membrane, playing a role in protecting and supporting insects (Merzendorfer, 2006; Zhu et al., 2016). Its synthesis, transformation, and modification are closely related to the growth and development of insects (Arakane et al., 2004; Arakane et al., 2005). Chitinase is involved in the degradation and resynthesis of chitin during the development of insect molting and metamorphosis. Its main function is to degrade chitin in insect tissue and is therefore an essential enzyme for insect growth and development (Qu et al., 2014; Muthukrishnan et al., 2019). Insect development from larva to adult requires repeated molting of old epidermis to form new epidermis, which is completed by the chitin synthesis and degradation pathway (Chen et al., 2008; Zhu et al., 2016). Trehalose metabolism affects chitin metabolism and an imbalance of trehalose metabolism have hindered chitin synthesis or degradation. The chitin synthesis pathway begins with trehalose and ends with chitin synthase, and both affect chitin synthesis during insect molting. When the expression of the *trehalase* gene was inhibited, the expression of related genes in chitin synthesis pathway and the *chitinase* gene also decreased, and chitin content was decreased, resulting in the obstruction of growth and development, difficulty in molting and death of insects (Merzendorfer and Zimoch, 2003; Tatun et al., 2008a; Tatun et al., 2008b; Chen et al., 2010; Tang et al., 2016; Jiang et al., 2022). Due to the physiological importance of trehalose in insects, it is regarded as a potential target for the study of insecticides, and such studies may lead to control strategies for certain insects (Tang et al., 2017; Tang et al., 2018; Matassini et al., 2020).

Nowadays, natural and effective trehalase inhibitors are mostly found in sugars, carbocyclic substances (Validamycin, Validoxylamines, Trehazolin, Salbostatin, etc.), and azasaccharides (Katagiri et al., 1998; Liebl et al., 2010; Matassini et al., 2020). These inhibitors can interact with the active site of trehalase, or compete with trehalose for the active site of trehalase, to inhibit the activity of trehalase (Tang et al., 2017). Validamycin A has a strong inhibitory effect on trehalase of *Rhizoctonia solani*. It can rapidly and effectively enter the bacterial cytoplasm and be hydrolyzed to produce Validamycin subunit amine A with stronger inhibitory effects on trehalase *in vivo* (Asano et al., 1987)*.* However, previous studies have shown that potential trehalose inhibitors, such as iminosaccharides, exhibit excellent specificity (Matassini et al., 2020). Candidates may lead to the development of an effective insecticide in the future. Therefore, the evaluation of new compounds remains significant.

*Spodoptera frugiperda*, belonging to Lepidoptera and Noctuidae, is an omnivorous pest native to tropical and subtropical America and an important agricultural pest (Todd and poole, 1980; Martinelli et al., 2006; Jia et al., 2022). The adult life span of *S. frugiperda* are 2–3 weeks, during which, female adults mate and lay eggs several times. The total number of eggs laid by a female in a lifetime range from 1, 500 to 2, 000. Under suitable temperatures, the eggs hatch into larvae within 2–10 days. Combined with its strong long-distance migration abilities (Pashley et al., 1985; Johnson, 1987; Wu et al., 2021), the rapid spread and serious harm caused by *S. frugiperda* has attracted global attention (Wang et al., 2022a). *Nature* published an article entitled “Invasive alien pests hit Africa hard,*”* which was about the large-scale invasion of *S. frugiperda,* causing global concern (Wild, 2017). Various measures have been taken to prevent and control the noctuid moth on the grassland, such as Bt maize (Quan and Wu, 2023). But so far, there is no means to comprehensively and effectively improve the overall control effects of *S. frugiperda*, due to its fast reproduction, wide harm, and strong migration ability, coupled with the lack of natural enemies, insufficient control experience, and poor control effects (Brévault et al., 2018; Kenis et al., 2022; Luo et al., 2022). Of cause, we known that residues of chemical pesticides caused environmental pollution and other problems (Desneux et al., 2007), so it is necessary to explore new environmentally friendly and effective means of pest control, for example, RNA insecticides (Nandety et al., 2015; Zhu and Pali, 2020; Yan et al., 2022), microbes (Liu et al., 2022), natural enemy insects or the combination of it and biological pesticides and so on (Kunte et al., 2020; Li et al., 2021; Wang et al., 2022b; Hou et al., 2022).

Aiming at the role of trehalase in insect glucose metabolism and the chitin metabolic pathway, we evaluated the inhibitory effects of validamycin and two potential inhibitory compounds, ZK-PI-5 and ZK-PI-9, on the growth and development of *S. frugiperda*, which may provide some ideas for the prevention and control of *S. frugiperda* (Luo et al., 2022). Those modified nucleoside analogues have shown potential activity as candidate insecticides (Hou et al., 2022).

## 2 Materials and methods

### 2.1 Experimental materials

The *S. frugiperda* was provided by the Zhejiang Academy of Agricultural Sciences (Hangzhou, China) and individuals were reared at Hangzhou Normal University. The larvae and adults were raised in an artificial climate box and room, respectively. Environmental parameters were set as follows: temperature 26°C ± 1°C; photoperiod 16:8 h (Light: Dark), and humidity 60% ± 10%. The larvae of *S. frugiperda* on the first day of the third instar were injected with trehalase inhibitor and three biological replicates were needed. Samples were taken 48 h after injection as samples for subsequent experiments. Furthermore, the potential inhibitory compounds ZK-PI-5 and ZK-PI-9 of trehalase evaluated were invented and provided by the PMDD Laboratory of China Agricultural University (Table 1; Supplementary Figure S1).

**TABLE 1:** Molecular formulas and relative molecular masses of ZK-PI-5 and ZK-PI-9.

Code	Amount/mg	Purity (%)	Solvent	MW	Molecular formula
ZK-PI-5	10	99	DMSO	337.4	C20H19NO4
ZK-PI-9	10	99	DMSO	341.8	C19H16ClNO3

### 2.2 Microinjection of trehalase inhibitor

Two potential inhibitory compounds, ZK-PI-5 and ZK-PI-9 were selected to 2 × 10^−3^ mmol/mL concentration to treat larvae after preliminary experimental screening. A group of 2%DMSO solvent was set as the control group (2%DMSO group). The larvae of *S. frugiperda* on the first day of the third instar were selected and injected 300 nL potential inhibitors and three biological replicates were needed. The needle was inserted into the thinner part between the second and third pair of thoracic feet of the larvae using a 3.5 mm diameter needle and TransferMan 4r (Eppendorf, Hamburg, German). Samples were taken 48 h after injection to determine trehalose, glucose, and glycogen content, trehalase activity, and related gene expression.

### 2.3 Determination of trehalase activity

The detection of trehalase activity was slightly modified according to Tatun’s method (Tatun et al., 2008a; Tatun et al., 2008b). The determination of enzyme activity required 30 *S. frugiperda* larvae as three biological replicates were needed. Firstly, after homogenization with stainless steel balls, 1 mL of phosphate buffered saline (PBS; pH = 7.0) was added for ultrasonic crushing for 30 min at 4°C and centrifuged at 10, 000 × g for 20 min. We then collected 350 μL of the supernatant, which was then ultracentrifuged at 4°C and 20, 800 × g for 60 min. The subsequent sample was used to determine soluble trehalase activity and protein concentration. The sediment was suspended at 300 μL in PBS, and the suspension was used to determine membrane-bound trehalase activity and protein concentration. Then, we combined a mixture of 60 μL of sample, 75 μL of 40 mM trehalose (Sigma-Aldrich, Saint Louis, Mo, United States), and 165 μL of PBS, which was then incubated at 37°C for 60 min, inactivated at 100°C for 5 min, and 130 μL of the mixture was placed in a water bath at 37°C for 30 min. The mixture of 130 μL was determined using a glucose (GO) detection kit (Sigma-Aldrich, Saint Louis, Mo, United States), and 260 μL was added after the reaction was terminated by 12 N H_2_SO_4_, the absorbance value was then measured at 540 nm.

### 2.4 Determination of sugar content

Here, the sample treatment before detection was conducted first. Thus, following homogenization, 1 mL PBS was added to the sample for ultrasonic crushing for 30 min and centrifugation at 4°C and 1,000 × g for 20 min. Subsequently, 350 μL of supernatant was ultracentrifugedat 4°C and 20,800 × g for 60 min, and the remaining supernatant was used to detect the contents of trehalose and glycogen. The supernatant and suspension, following ultracentrifugation, were used to determine the glucose content.

In the second step, trehalose content was detected by the anthrone method (Leyva et al., 2008) where 1% sulfuric acid was added to 30 μL of the sample, which was then placed in a 90°C water bath for 10 min, and an ice bath for 3 min. Subsequently, we added 30 μL of 30% KOH, and the sample was placed in a 90°C water bath for 10 min, then an ice bath for 3 min, after which 600 μL of developer (0.02 g of anthrone +100 mL of 80% H_2_SO_4_) was added and the sample was placed in a 90°C water bath for 10 min and cooled in an ice bath. The absorbance was then detected at 630 nm. In the third step, the detection methods of glycogen and glucose are similar, except that trehalose was aliquoted 160 μL and 32 μL of 0.1 U/L amylo-transglucosidase was added, the mixture was then subjected to a 40°C water bath for 4 h to convert it into glucose. Next, we used the glucose (GO detection kit to determine the content of substances in the mixed solution, and then added 12 N H_2_SO_4_ to terminate the reaction. The absorbance value was detected at 540 nm.

### 2.5 RNA extraction and cDNA synthesis

First, the samples were pretreated. Parallel sampling was adopted in the experiment, that is, they were divided into three treatment groups, with 3 larvae in each group injected for 48 h, then placed in the freezer at −80°C. All the sample was added to a centrifuge tube containing 200 μL of Trizol (Invitrogen, Carlsbad, California, United States), after which the sample was fully ground with an electric homogenizer, and made up to 1 mL with Trizol. Subsequently, 200 μL chloroform was added, and the sample was centrifuged at 4°C and 12, 000 rpm, for 10 min to obtain the supernatant. Then 500 μL of isopropyl alcohol was added to the sample and the previous centrifugation step was repeated. Subsequently, the supernatant was discarded, 1 mL of 75% ethanol was added, and the precipitate was blown away from the pipe wall. The precipitate was washed and centrifuged at 4°C and 7, 500 rpm for 5 min. The sample was then centrifuged at 4°C and 12, 000 rpm for 10 min and ethanol was added. Finally, 30–50 μL DEPC of treated water was dissolved and precipitated and for each sample, 1 μL of RNA was used for electrophoretic and spectrophotometer concentration detection, respectively.

Using a NanoDrop™2000 (Thermo Fisher Scientific, New York, NY, United States), we determined the concentration and purity of total RNA extracted using a PrimeScript^®^RT reagent Kit with a gDNA Eraser (Takara, Japan) reaction system to ensure that the total amount of RNA added in each sample reverse transcription system was equal. Finally, the first strand of cDNA was reverse transcribed using the PrimeScript^®^ RT reagent Kit with a gDNA Eraser reverse transcription kit according to the manufacturer’s instructions.

### 2.6 RT-qPCR

The specificity, concentration, and annealing temperatures of the primers (Table 2) were explored, and the optimum amount and temperature of primers in the reaction system were obtained by comparative analysis. Ribosomal protein L10 (RPL10) was as internal reference gene. The gene expressions of *TRE1*, *TRE2*, *CHS2,* and *CHT* were detected by real-time fluorescence quantitative PCR using 10.0 μL of the PCR reaction system, 5 μL of SYBR Premix Ex Taq (Takara, Japan), 0.4 μL of forward primer (10 pmol), and 0.4 μL of reverse primer (10 pmol), 1 μL of template cDNA, and 3.2 μL of RNase free ddH_2_O. The reaction procedure included pre-denaturation at 95°C for 2 s, denaturation at 95°C for 30 s, and annealing at 59°C for 30 s (35 cycles). Finally, the melting curve was drawn at 65°C–95°C.

**TABLE 2:** Primer sequences of *S. frugiperda*.

Primer	Forward primer sequence (5′-3′)	Reverse primer sequence (5′-3′)
*SfTRE1*	TCA​GAT​GAA​GGT​GAA​CTC​GAA​GA	GGA​ATG​ATG​AAT​CCG​TGG​GTA
*SfTRE2*	CTG​CTG​CTG​TCG​GAG​ATG​A	TAG​GAG​GGG​AGG​CTG​TGA​T
*SfCHS*	GAG​TTC​ACA​GTG​CGG​TTG​C	GCC​AAA​ATA​GCC​CAC​ATC​C
*SfCHT*	AAGCGGACAGCAAAGCG	CCA​ACT​CAG​GGT​CAA​TAA​TAA​GAA​C
*qRT-RPL10*	GACTTGGGTAAGAAGAAG	GATGACATGGAATGGATG

### 2.7 Determination of chitin content and chitinase activity

Before the experiment, the samples were pretreated. The samples were divided into three aliquots and poured into liquid nitrogen for grinding. After mixing with the KOH solution at 4°C, the samples were placed in a water bath at 80°C for 90 min, and then centrifuged at 12, 000 × g at 4°C for 20 min and the precipitates were obtained. They were resuspended in 1 mL of PBS buffer, centrifuged at 12, 000 × g at 4°C for 20 min, and resuspended at 200 μL of McIlvaine’s buffer (0.1 mol/L citric acid and 0.2 mol/L NaH_2_PO_2_, pH = 6). The supernatant was then isolated and 5 μL of chitinase from *Streptomyces griseus* (Sigma-Aldrich, Saint Louis, MO, United States) was added, the sample was then placed in a warm water bath at 37°C for 72 h, prior to testing.

The solution to be tested was centrifuged at 12, 000 × g for 1 min at room temperature, and three 60 μL aliquots of samples are isolated. We added 60 μL of supernatant to each, then 0.27 mol/L sodium borate, evenly mixed the solution at RT, and centrifuged it at 12, 000 × g for 1 min. Subsequently, the samples were heated in a 99.9°C thermal circulator for 60 s, and then mixed in a 99.9°C water bath for 10 min, and cooled to RT with ice. Finally, to the sample, we added 600 μL of 1×DMAB and determined the absorbance value at a wavelength of 585 nm, this was triplicated and finally the average value was calculated to determine chitin content. Using the Chitinase Kit (Suzhou Comin Biotechnology Co., Ltd., China) according to the manufacturer’s instructions, we weighed 0.1 g of tissue to extract crude enzyme solution, set the experimental and control groups for experiment, measured the light absorption value at 540 nm, and finally calculated chitinase activity.

### 2.8 Statistics on growth and development of *S. frugiperda*

For this experiment we set up a control group (about 150 untreated third instar larvae of *S. frugiperda*). Other samples were microinjected with ZK-PI-5 and ZK-PI-9. A hundred and fifty larvae were injected respectively, and 50 larvae in parallel. From the first day after the injection of trehalase inhibitor, the larval age was recorded daily and compared with the control group. We also monitored the molting of larvae daily, recorded the number of normal and abnormal larvae respectively, took photos of abnormal larvae to observe phenotypic changes, measured the body length with a ruler, registered the number of deaths, and entered the mortality into the statistical table.

In addition, more than 30 larvae were taken from the treatment and control groups respectively, 10 larvae were treated in parallel, and tests were triplicated. The *S. frugiperda* pupa were weighed, their length was measured, and the pupation rate was recorded following injection. The emergence rate was recorded following adult eclosion. If the adult could not come out or did not have normal wings during eclosion, it was considered to be abnormal. If the adult did not appear after 30 days, it was considered dead, and all deformed individuals were photographed and recorded by camera (Canon, EOS50D, Japan).

### 2.9 Data analysis

Excel software 2020 and IBM SPSS Statistics 8.0 were used for statistical analysis. One way ANOVA method or *t*-test were used to determine statistically significant differences, and the final values were expressed as mean ± standard error. The relative expression of the target gene was calculated by 2-^△△ CT^ (Livak and Schmittgen, 2001).

## 3 Results

### 3.1 Changes in trehalase activity of *S. frugiperda* after injection of trehalase inhibitor

**FIGURE 1:**
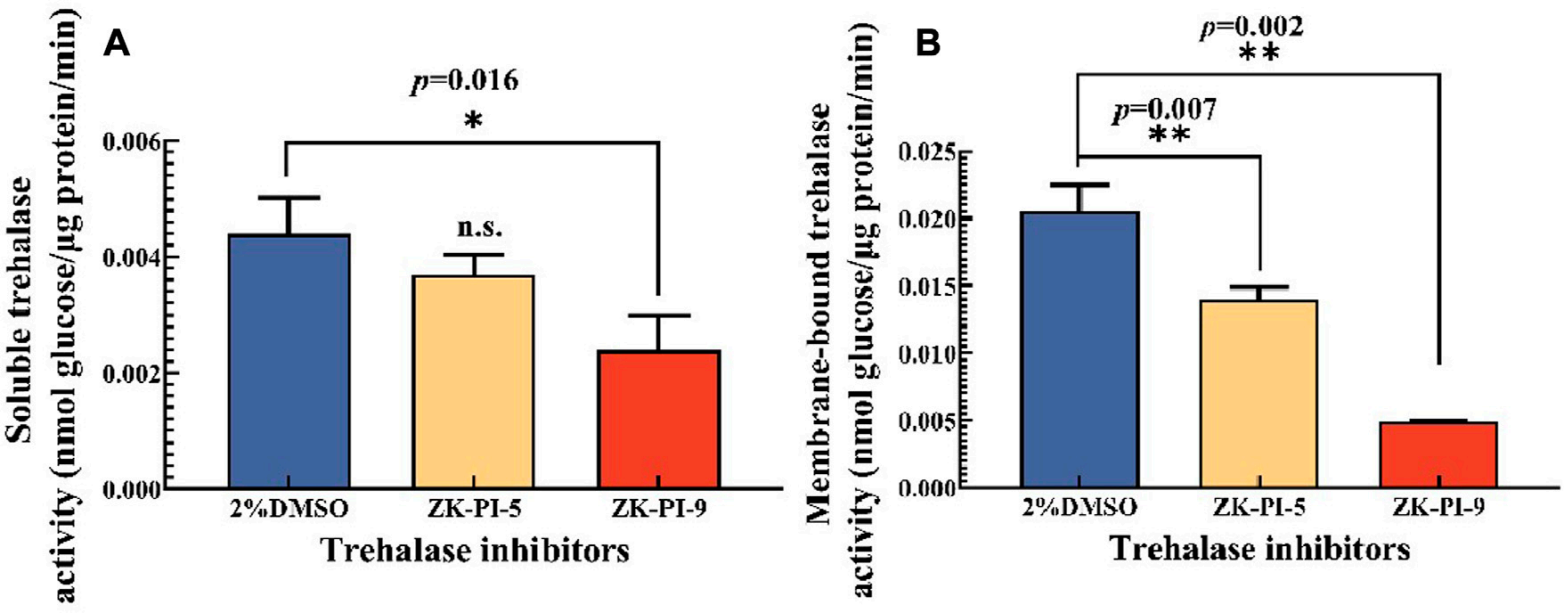
Changes in trehalase activity 48 h after injecting two trehalase inhibitors. **(A)** Change in soluble trehalase activity; **(B)** Changes in membrane-bound trehalase. The *S*. *frugiderda* larvae on the first day of the third instar were divided into three parallel groups, injected with two trehalase inhibitors or left untreated, and used to detect trehalase activity after 48 h. Values are presented as the means ± SE. *p < 0.05 denotes significant differences. **p < 0.01 denotes extremely significant differences, n.s.: not significant (independent-samples *t*-test).

rehalase is the only hydrolase that decomposes trehalose. We found that the activities of soluble and membrane-bound trehalase were significantly inhibited after injecting ZK-PI-9 into the larvae, and the enzyme activities were 45.3% and 76.2% lower than the 2%DMSO group respectively (Figure 1). In contrast, there was no significant change in soluble trehalase activity but inhibited membrane-bound trehalase activity (31.7% lower than the 2%DMSO group) after ZK-PI-5 treatment (Figure 1).

### 3.2 Changes in glycogen, trehalose, and glucose in *S. frugiperda* after injection of trehalase inhibitor

**FIGURE 2:**
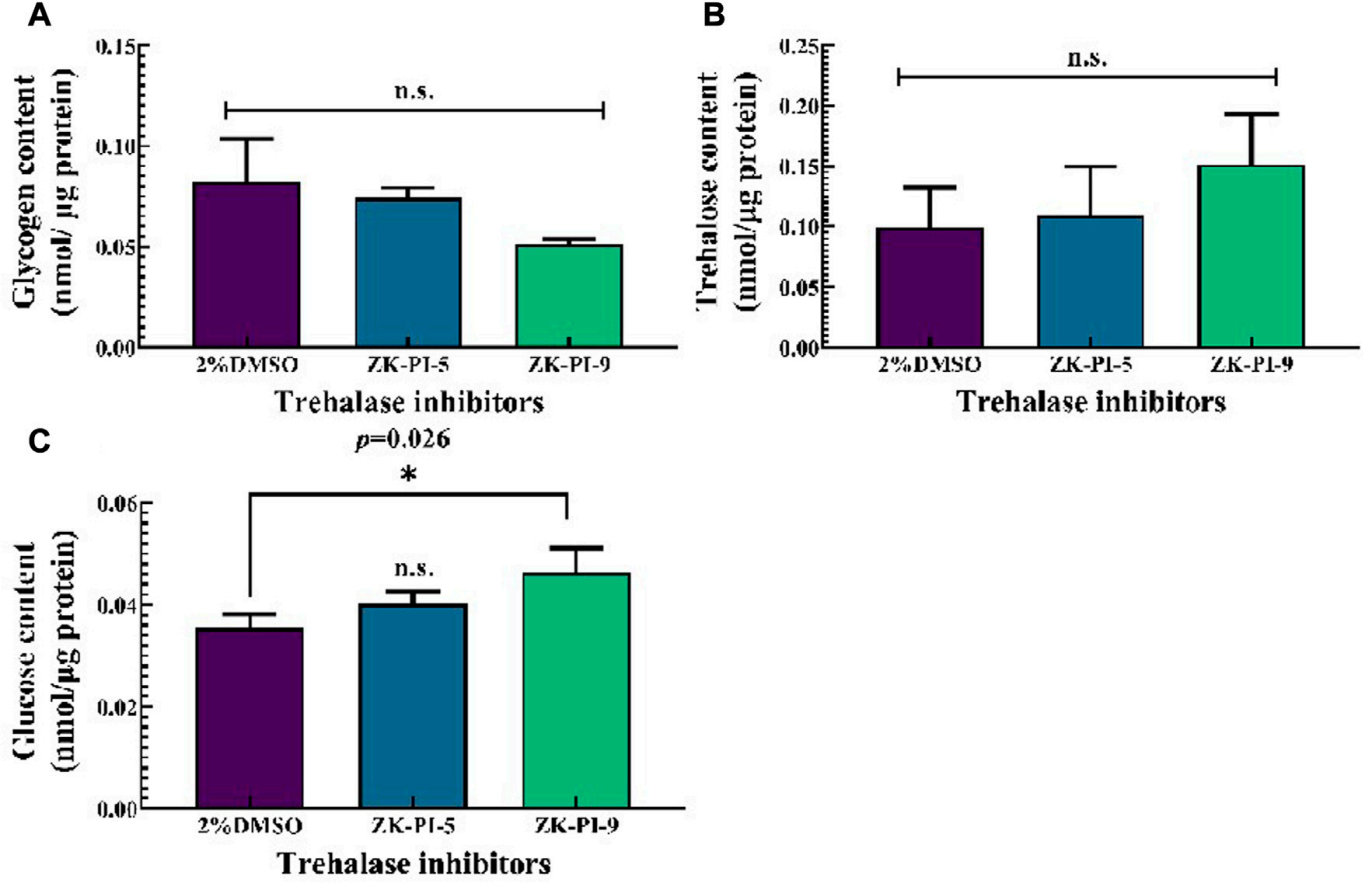
Changes in trehalose **(A)**, glucose **(B)** and glycogen content **(C)** 48 h after injecting two trehalase inhibitors. The *S*. *frugiderda* larvae on the first day of the third instar were divided into three parallel groups, injected with two trehalase inhibitors or left untreated, and used to detect the content of the 3 sugars after 48 h. Values are presented as the means ± SE. *p < 0.05 denotes significant differences. **p < 0.01 denotes extremely significant differences, n.s.: not significant (independent-samples *t*-test).

Compared with the control group (2%DMSO group), ZK-PI-5 and ZK-PI-9 had no significant effect on the glycogen content (Figure 2A). There was also no significant difference in trehalose content between groups injected with the two inhibitors and the 2%DMSO group (Figure 2B). Compared with the 2%DMSO group, Glucose content significantly increased after ZK-PI-9 treatment, but the effects of ZK-PI-5 treatment on glucose content was not evident (Figure 2C).

### 3.3 Changes in trehalose and chitin metabolism related gene expression after injection of trehalase inhibitor

**FIGURE 3:**
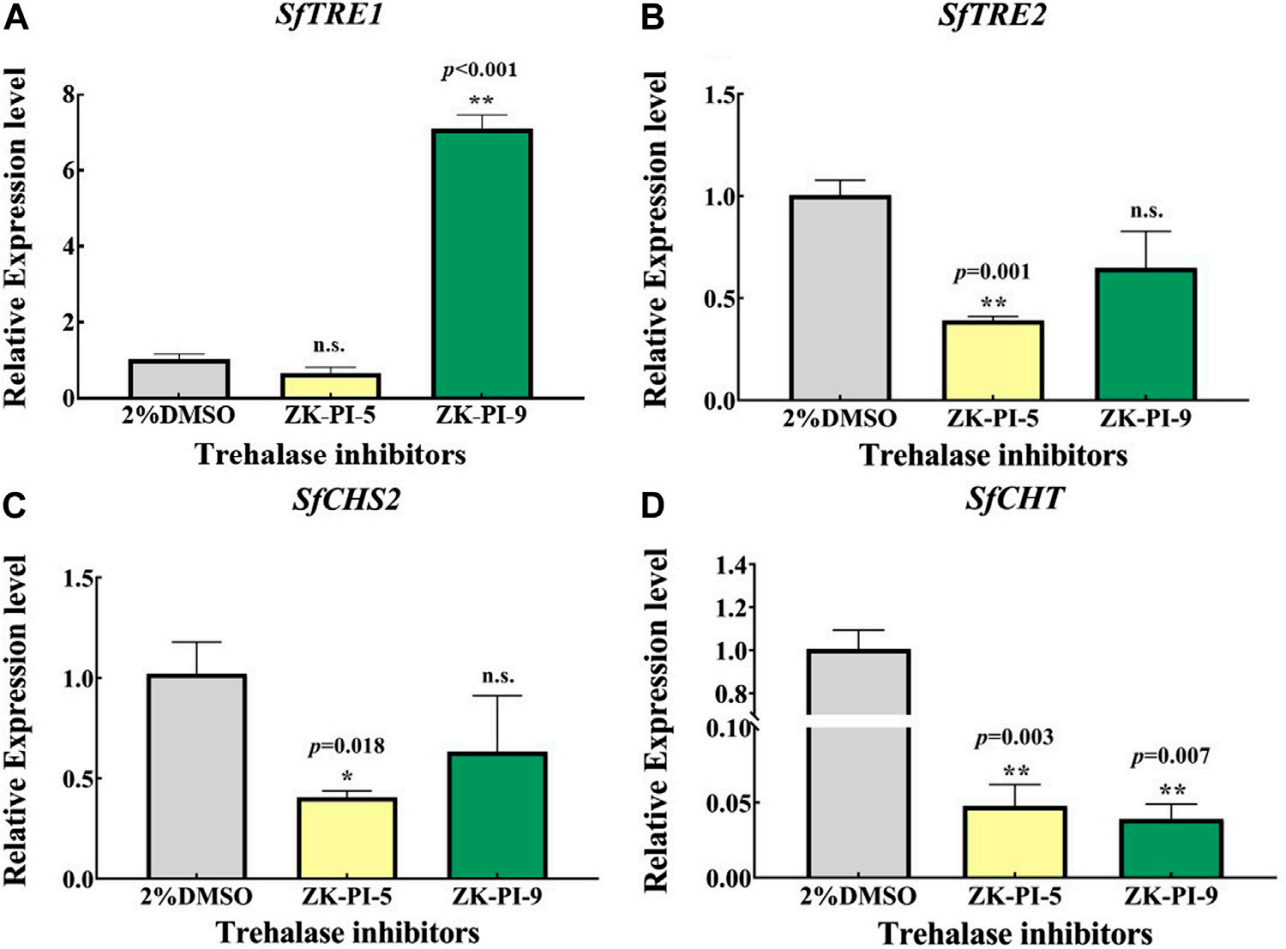
The mRNA relative expression levels of *TRE1* **(A)**, *TRE2* **(B)**, *CHS2* **(C)**, and *CHT* **(D)** genes in *S. frugiderda* after injection of both trehalase inhibitors over 48 h. Expression of two trehalose metabolism pathway genes **(A,B)** and two chitin metabolism pathway genes **(C,D)** after 48 h, levels were measured using q-RT-PCR. *TRE1*, soluble trehalase; *TRE2*, membrane-bound trehalase; *CHS2*, chitin synthase 2; *CHT*, chitinase. Values are presented as the means ± SE. *p < 0.05 denotes significant differences. **p < 0.01 denotes extremely significant differences, n.s.: not significant (independent-samples *t*-test).

3*TRE1* and *TRE2* are the key genes of trehalose metabolism, while *CHS* and *CHT* are the key genes of chitin metabolism. We detected the mRNA expression levels of gene transcripts in *S. frugiperda* after injection of the inhibitor. The results showed that the expression of *SfTRE1* gene was significantly higher but the expression of *SfCHT* gene was significantly lower after injection with ZK-PI-9 than the control group (Figures 3A, D). In addition, the expression of *SfTRE2*, *SfCHS2*, and *SfCHT* decreased significantly after injection of ZK-PI-5 (Figures 3B–D).

### 3.4 Changes in chitin content and chitinase activity in *S. frugiperda* after injection of trehalase inhibitor

**FIGURE 4:**
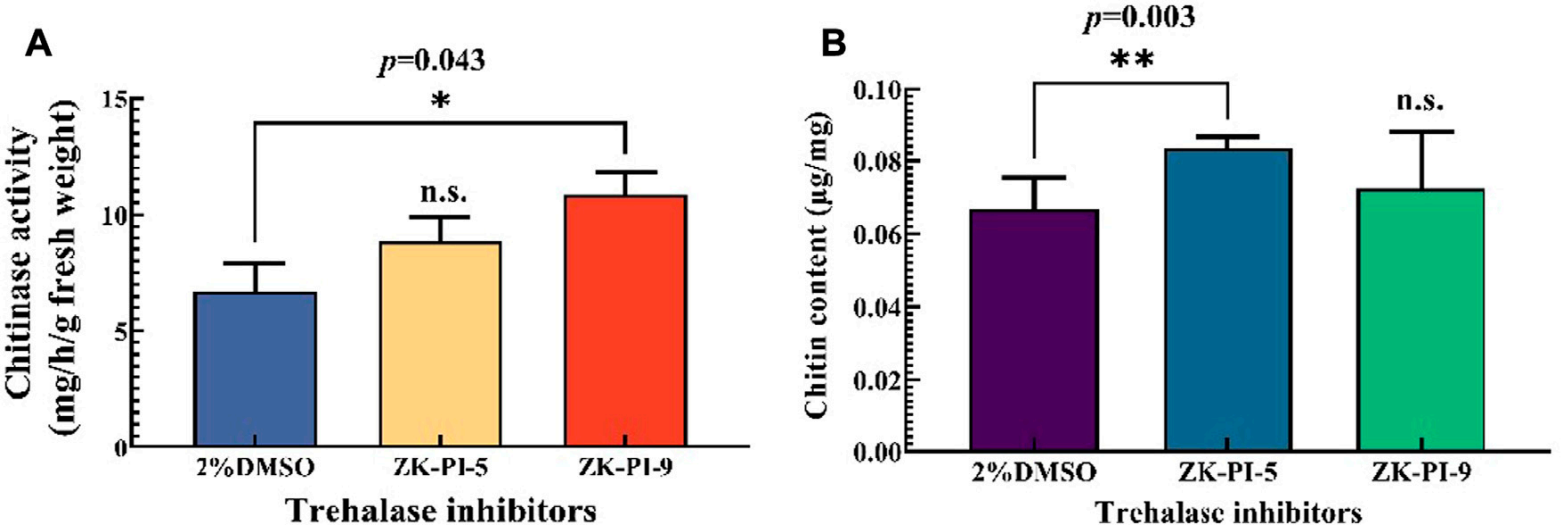
Changes in chitinase activity **(A)** and chitin content **(B)** 48 h after injection of two trehalase inhibitors. Values are presented as the means ± SE. *p < 0.05 denotes significant differences. **p < 0.01 denotes extremely significant differences, n.s.: not significant (independent-samples *t*-test).

Chitinase activity in larvae increased significantly, particularly following ZK-PI-9 injection (Figure 4A). Compared with the control group (2%DMSO group), the chitin content of the larvae injected with ZK-PI-5 increased significantly. However, there was no significant difference in chitin content between groups injected with ZK-PI-9 and the 2%DMSO group (Figure 4B).

### 3.5 Changes of larval length and development duration of *S. frugiperda* after injection of trehalase inhibitor

By measuring the larva body length of *S. frugiperda*, we found that the length of the fourth instar and fifth instar larvae of *S. frugiperda* was significantly higher in the ZK-PI-5 group than the control group, however, there was no significant change in sixth instar larva length. Besides, there was no significant change in the ZK-PI-9 group compared with control group (Table 3). According to observation, the developmental time of *S. frugiperda* showed that there was little difference in some instars of *S. frugiperda* injected with ZK-PI-5 and ZK-PI-9 trehalase inhibitors, but there was no significant difference in the total developmental period from fourth instar to pupal stage (Table 4).

**TABLE 3:** Length of *S. frugiperda* larvae.

Unit: cm	4th instar	5th instar	6th instar
2%DMSO	1.55 ± 0.033 b	2.35 ± 0.047 b	2.81 ± 0.067 a
ZK-PI-5	1.76 ± 0.048 a	2.55 ± 0.065 a	2.62 ± 0.065 a
ZK-PI-9	1.65 ± 0.015 ab	2.27 ± 0.034 b	2.75 ± 0.087 a

Comparisons were made between three different treatments at the same larval stage of *S. frugiperda*. Different letters denote significant difference from the control (2%DMSO, group) at 0.05 level, respectively. Values are presented as the means ± SE (ANOVA, followed by Tukey’s *post hoc* test).

**TABLE 4:** Developmental duration of *S. frugiperda*.

Unit: day	4th instar	5th instar	6th instar	Pre-pupa	Pupa	Developmental duration (4th instar to pupa)
2%DMSO	2.57 ± 0.18 a	1.47 ± 0.15 b	1.57 ± 0.18 ab	1.2 ± 0.12 b	7.64 ± 0.16 a	14.73 ± 0.333 a
ZK-PI-5	2.11 ± 0.12 a	1.22 ± 0.08 b	1.89 ± 0.17 a	1.22 ± 0.12 b	7.25 ± 0.13 a	13.58 ± 0.288 a
ZK-PI-9	1.79 ± 0.35 a	2.21 ± 0.14 a	1.11 ± 0.12 b	1.78 ± 0.08 a	7.25 ± 0.13 a	13.67 ± 0.512 a

Different letters denote significant difference from the control (2%DMSO, group) at 0.05 level, respectively. Values are presented as the means ± SE (ANOVA, followed by Tukey’s *post hoc* test).

### 3.6 Changes in pupal weight and length of *S. frugiperda* after injection of trehalase inhibitor

**FIGURE 5:**
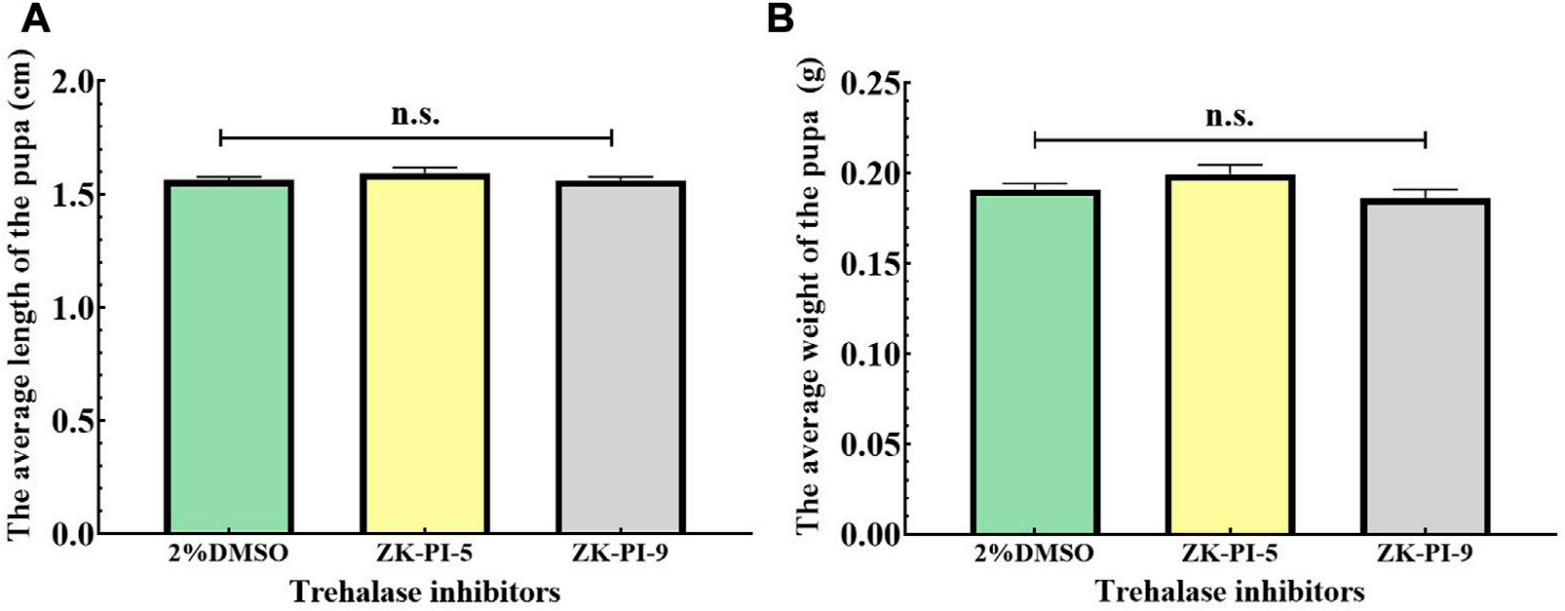
Pupal length **(A)** and weight **(B)** of *S. frugiderda* after injection of two trehalase inhibitors. The body length and weight of *S. frugiderda* in pupal stages were measured and recorded. Values are presented as the means ± SE. n.s.: not significant (independent-samples *t*-test).

The pupal length of *S. frugiperda* injected with ZK-PI-9 and ZK-PI-5 trehalase inhibitor has no significant effect compared with 2%DMSO pupal length (Figure 5A). In addition, there were also no significant difference in weight of *S. frugiperda* after ZK-PI-5 and ZK-PI-9 injection (Figure 5B).

### 3.7 Changes of pupation rate and emergence rate after injection of trehalase inhibitor

**FIGURE 6:**
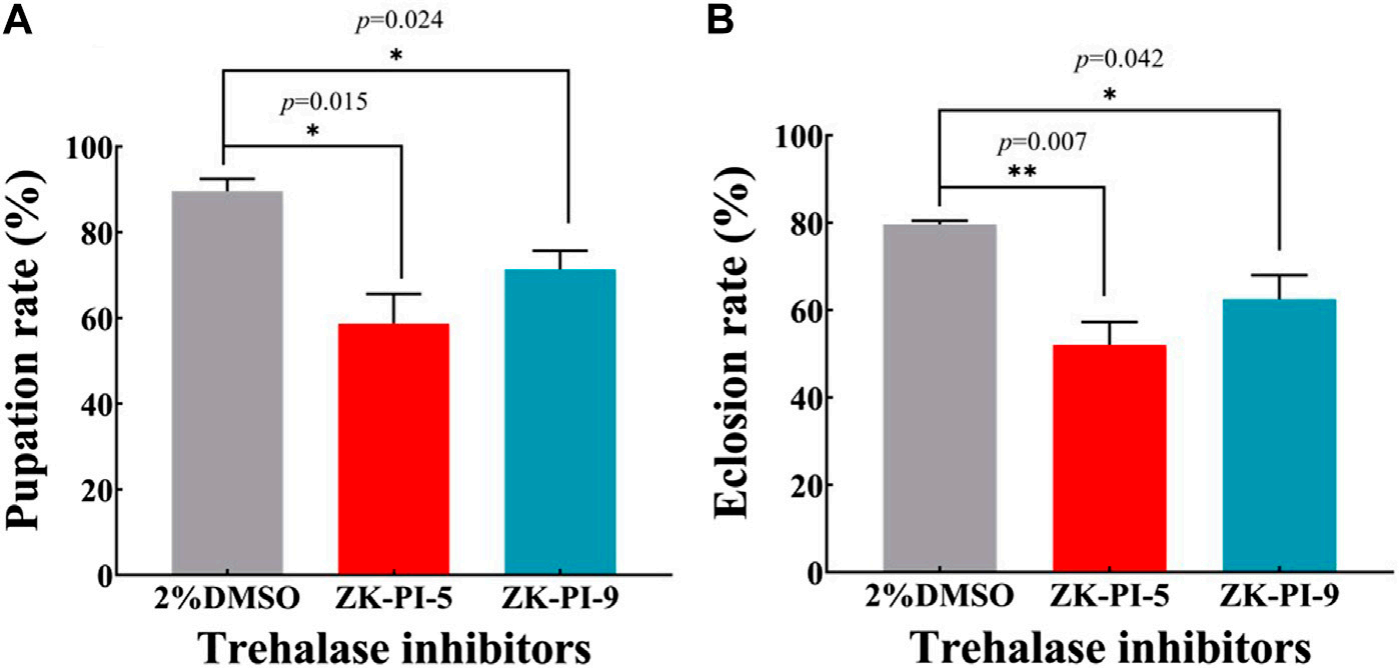
The pupation **(A)** and eclosion rates **(B)** of *S. frugiderda* after injection of two trehalase inhibitors. The changes in pupation and eclosion rates during the development of *S. frugiderda* after injection of trehalase inhibitors were recorded compared with the control group. Pupation rate = (pupal number/6th instar larval number) * 100%; Eclosion rate = (number of adults/pupal number) * 100%. *N* = 150 per treatment. Values are presented as the means ± SE. *p < 0.05 denotes significant differences. **p < 0.01 denotes extremely significant differences (independent-samples *t*-test).

Compared with the control group (2%DMSO group), the pupation rate of *S. frugiperda* injected with ZK-PI-5 and ZK-PI-9 trehalase inhibitors decreased significantly by 34.5% and 20.4% respectively (Figure 6A). Furthermore, after ZK-PI-5 and ZK-PI-9 injection, the eclosion rate of the ZK-PI-5 and ZK-PI-9 group all were significantly lower than that of the control group by 34.7% and 20.6% respectively (Figure 6B).

### 3.8 Changes of mortality and death phenotype of *S. frugiperda* after injection of trehalase inhibitor

**FIGURE 7:**
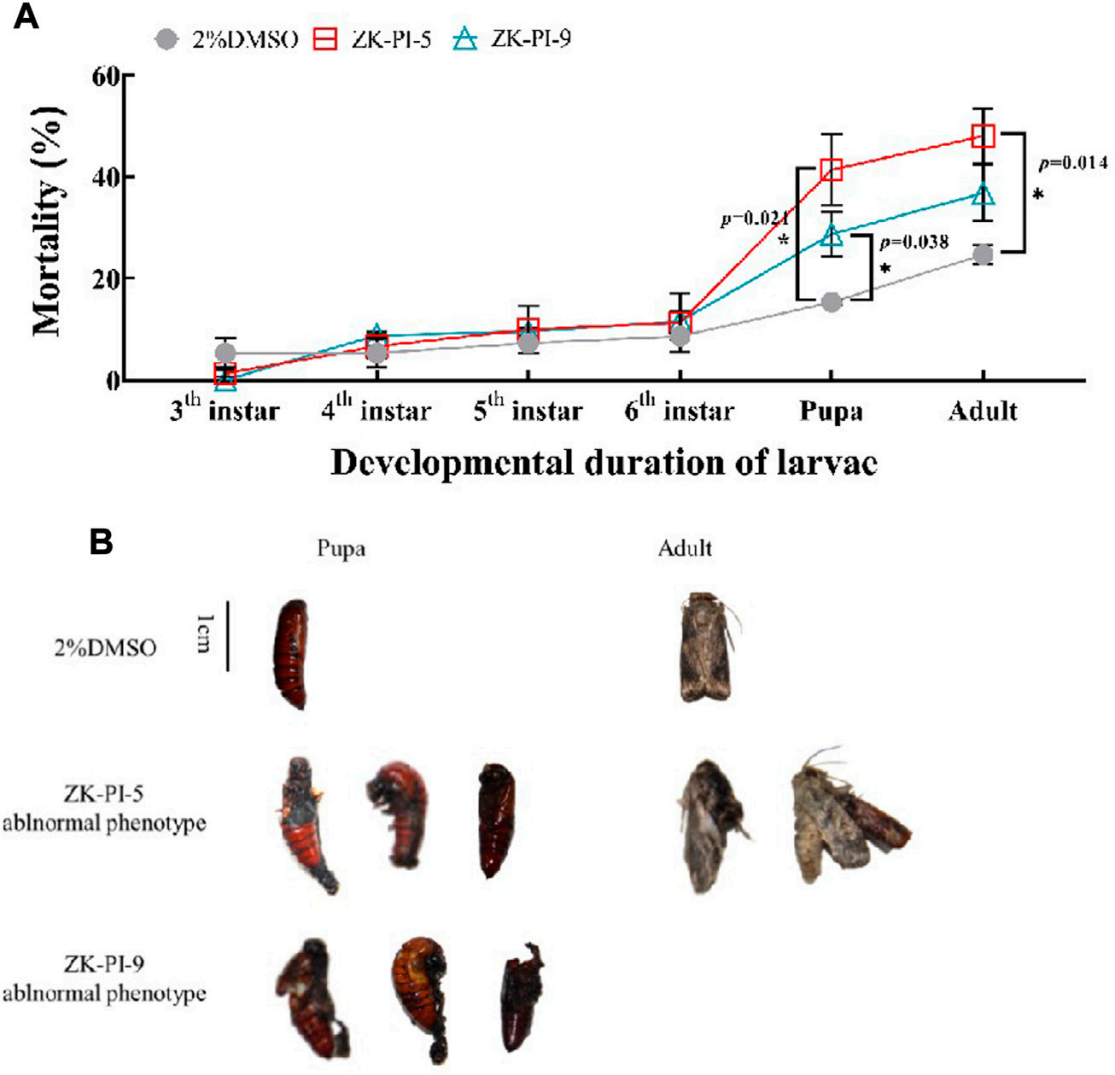
Death rates **(A)** and phenotype **(B)** of *S. frugiderda* after injection of two trehalase inhibitors. The number of deaths of *S. frugiderda* during development after injection of trehalase inhibitors were counted, and all death phenotypes were recorded. Values are presented as the means ± SE. *p < 0.05 denotes significant differences. **p < 0.01 denotes extremely significant differences (independent-samples *t*-test).

Results showed that *S. frugiperda* injected with ZK-PI-5 had significantly higher mortalities during the pupation and eclosion stages than the 2%DMSO group (Figure 7A). After injection of ZK-PI-9, *S. frugiperda* had only significantly higher mortalities at the pupation. In general, both two trehalase inhibitors increased the mortality rates of *S. frugiperda*, particularly during pupation and eclosion. In addition, we found that ZK-PI-5 and ZK-PI-9 caused different death phenotypes of *S. frugiperda* during pupation and eclosion. The pupal phenotype included molting failure and abnormal pupation, and the adult phenotype included wing deformity and an inability to break pupae (Figure 7B). These results showed that ZK-PI-5 and ZK-PI-9 caused the death of *S. frugiperda* due to abnormal development, and the effect was significant. In general, ZK-PI-5 has a more significantly lethal effect.

## 4 Discussion

In this study, ZK-PI-5 and ZK-PI-9 were compared to explore the potential inhibitory abilities to trehalase. The results showed that ZK-PI-9 had obvious inhibitory effects on trehalase activity, and the activities of soluble and membrane-bound trehalase decreased significantly after ZK-PI-9 injection (Figure 1). Studies showed that validamycin, a trehalase inhibitor, could “compete” with trehalose for trehalase, thus, inhibiting trehalase and affecting insect growth and development (Asano et al., 1987; Zhu and Palli, 2020; Yan et al., 2022); other trehalase inhibitors have the same functions (Shukla et al., 2015; Kunte et al., 2020; Liu et al., 2022). It represented that ZK-PI-9 has similar inhibitory effects as validamycin and is a high-efficiency trehalase inhibitor. Moreover, it was assumed that ZK-PI-5 was an effect membrane-bound trehalase inhibitor and resulted in decreased membrane-bound trehalase activity (Figure 1).

We found no significant difference in glycogen content and trehalose content between ZK-PI-5 and ZK-PI-9 treatment and the group in this study. While past studies have shown that trehalose is hydrolyzed by trehalase to produce glucose (Li et al., 2021), and glucose on uridine diphosphate glucose completes the last step of glycogen synthesis under the action of glycogen synthase (Wang et al., 2020; Liu et al., 2022). Through past experiments, we also found that trehalase activity inhibition have prevented trehalose from converting to glucose, and glucose content has been decreased. Glycogen synthase catalyzes UDP-glucose to glycogen. *Spodoptera frugiperda* might be feeding as possible when stimulated in high validamycin concentration to promote glycogen synthesis. Glycogen, trehalose, and glucose could transform each other to jointly maintain the trehalose concentrations (Kameda et al., 1987; Leyva et al., 2008; Luo et al., 2022). In most studies, typical trehalase inhibitor, validamycin or validoxylamine A, have increased trehalose content and decreased glucose content. But our results have showed that glucose content in the ZK-PI-9 group increased significantly (Figure 2C). Moreover, ZK-PI-9 inhibited trehalase activity increased chitinase activity (Figure 4A). Interestingly, soluble trehalase activity decreased but expression level of *SfTRE1* was upregulated (Figure 3A), similarly, chitinase activity increased but expression level of *SfCHT* was downregulated (Figure 3D), it seems to be a mechanism to maintain physiological and metabolic stability *in vivo*. These results were not completely consistent with changes caused by typical trehalase inhibitors (Tatun et al., 2014; Shukla et al., 2015), thus we suspected that the negative effect of ZK-PI-9 on *S. frugiperda* was not obvious compared with ZK-PI-5.

The key genes, *SfTRE2*, *SfCHS2,* and *SfCHT,* involved in trehalose and chitin metabolism of *S. frugiperda* were affected by ZK-PI-5 (Figure 3). In this study, compared with 2%DMSO group, the mRNA expression of *SfTRE2* in ZK-PI-5 group decreased significantly (Figure 3B), indicating that ZK-PI-5 not only inhibited membrane-bound trehalase activity but also downregulated the expression of membrane-bound trehalase gene. Moreover, it even effectively downregulated the expression of *SfCHS2* and *SfCHT* (Figures 3C, D), which was consistent with Silva et al. (2010) and Zhang et al. (2017). Previous studies have shown that changes in trehalase gene expression level affect chitin biosynthesis in insects (Shukla et al., 2015; Tang et al., 2017). It was found that ZK-PI-5 treatment enhanced chitin content compared with 2%DMSO group. It is known that *TcCHS2* downregulation only affects chitin synthesis of peritrophic membranes (Xia et al., 2002), which was a small fraction of the total chitin content. Accordingly, the decreased expression level of *SfCHT* may be responsible for increased chitin content.

Chitin is an important component of the epidermis, which is closely related to the growth and development of insects (Merzendorfer, 2006; Merzendorfer, 2011). Trehalase has a significant effect on chitin synthesis during insect molting (Merzendorfer and Zimoch, 2003; Tatun et al., 2008b; Chen et al., 2010). It was believed that *SeTRE-1* played a major role in *CHSA* expression and chitin synthesis in the stratum corneum, and *SeTRE-2* played an important role in *CHSB* expression and chitin synthesis in the midgut (Chen et al., 2010). *CHS1* silencing in *Lepeophtheirus salmonis* led to incomplete molting or abnormal cuticles (Roach et al., 2012), and the growth and development of beet armyworm was inhibited and abnormal formation of epidermal chitin layer as well as trachea were occuredafter *CHS1* RNAi (Contreras et al., 2016). The *TcCHS1* inhibition in *Tribolium castaneum* affected each molt of the insect (Iordachescu and Imai, 2008). When *TcCHS2* was inhibited, chitin in the adult peritrophic membranealmost disappeared, embryonic development was also affected, and female oviposition rate decreased. Interfering with *LmCHS2* transcription of *Locusta migratoria* reduced midgut chitin synthesis, resulting in high mortality of the insect (Liu et al., 2012). The above studies have showed that changes in *CHS* gene expression led to the obstruction of insect development, deformity, molting difficulty, and even death, thereby affecting insect growth and development (Roach et al., 2012; Tang et al., 2012; Contreras et al., 2016; Harðardóttir et al., 2021). We confirmed that ZK-PI-5 could directly reduce membrane-bound trehalase activity, and downregulate the expression level of *SfTRE2* and *SfCHS2*, lead to chitin metabolism disorder, affect chitin content and the growth and development of *S. frugiperda*, and lead to abnormal development and even death of *S. frugiperda* (Figure 7B). It is therefore speculated that trehalase silencing may affect the regulation of chitin biosynthesis and degradation, resulting in insect pupation or eclosion deformity.

The growth and development of *S. frugiperda* was affected by trehalose. Trehalose can provide energy for insects and plays an important role in energy metabolism and stress resistance (Leyva et al., 2008). The trehalase inhibitor inhibited the function of trehalase. Studies have shown that trehalase inhibitors can affect a series of physiological functions in insects, including chitin synthesis, stress protection, and larval and pupal development (Chen et al., 2010; Liebl et al., 2010; Shen et al., 2017; Liu et al., 2022; Yan et al., 2022). The injection of trehalase inhibitors, such as validamycin A and trehazolin, into insect larvae have resulted in abnormal molting of larvae and the failure of pupation or fatal metamorphosis of adults. For example, the inhibition of trehalose metabolism interferes with the normal chitin synthesis of locusts and increases deformity rates (Wegener et al., 2003; Zhao et al., 2016; Li et al., 2021). In our research, the injection of ZK-PI-5 and ZK-PI-9 greatly increased the mortality of *S. frugiperda* during pupation and eclosion. Combined with the death phenotype of *S. frugiperda* (Figure 7B) and the development time (Table 3), particularly ZK-PI-5 inhibitors had a significantly inhibitory effect on the growth and development of *S. frugiperda*. It was very likely that the presence of inhibitors significantly downregulated the expression level of *SfTRE2* and *SfCHS2*, thus, inhibiting chitin metabolism. In addition, according to the abnormal death of eclosion in the ZK-PI-5 group and the death phenotype in the ZK-PI-9 group, we speculated that the trehalase inhibitor affected trehalose metabolism, causing *S. frugiperda* to not have enough energy to support its pupation and eclosion, which was consistent with the results of previous research on trehalase inhibitor.

## 5 Conciusion

This experiment was carried out on a trehalose generation of *S. frugiperda* to determine the content of related substances, enzyme activity, and gene expression levels during trehalose and chitin metabolism, combined with the relevant data of the growth and development of *S. frugiperda*, to explore the inhibitory effects of ZK-PI-5 and ZK-PI-9 inhibitors on *S. frugiperda*. ZK-PI-9 was highly effective at inhibiting trehalase activity and ZK-PI-5 and ZK-PI-9 significantly inhibited the expression of key genes of chitin metabolism of *S. frugiperda*. The determination of the death phenotype, pupation and emergence rates of *S. frugiperda* confirmed the inhibitory effects of ZK-PI-5 and ZK-PI-9 on chitin metabolism, showing that ZK-PI-5 had a more significant effect. Thus, ZK-PI-9 is a trehalase inhibitor, and ZK-PI-5 tends to directly affect insect trehalose and chitin metabolism as a specific membrane-bound trehalase inhibitor.

This study provides data support for the inhibitory effects of ZK-PI-5 and ZK-PI-9. Based on the dependence of insects on trehalose, the inhibitory effects of ZK-PI-5 on membrane-bound trehalase was more specific, had a more prominent impact on the measured indexes, and is therefore a more suitable trehalase inhibitor. This provides a theoretical basis for the application of ZK-PI-5 as a control for *S. frugiperda* and a foundation for the research and development of more effective biological pesticides (although more research would be needed to assessing potential impact on *S. frugiperda* population growth, e.g., using demographic analytical methods, see Chi et al., 2020; Chi et al., 2022a; Chi et al., 2022b). However, more in-depth exploration is still needed to determine how ZK-PI-5 trehalase inhibitor can effectively be used in the research and development of biological pesticides and whether it can be produced on a large scale.

## Data Availability

The original contributions presented in the study are included in the article/Supplementary Material, further inquiries can be directed to the corresponding authors.
