# Supplementary material for: Potential inhibitory effects of compounds ZK-PI-5 and ZK-PI-9 on trehalose and chitin metabolism in Spodoptera frugiperda (J. E. Smith)
Source: Front Physiol. 2023 Mar 29;14:1178996. doi: 10.3389/fphys.2023.1178996 (PMC10090375; doi:10.3389/fphys.2023.1178996)
Supplement: Supplementary file 1 [file Table1.DOCX]

Supplemental files

S1. Structural formulas of ZK-PI-5 and ZK-PI-9
